# List of the names of the referees for the studies that were published in the São Paulo Medical Journal/Evidence for Health Care during the year 2010

**DOI:** 10.1590/S1516-31802010000600015

**Published:** 2010-12-02

**Acary Souza Bulle Oliveira** - Universidade Federal de São Paulo (Unifesp)
**Afonso Celso Pinto Nazário** - Universidade Federal de São Paulo (Unifesp)
**Alberto Allain Gabbai** - Universidade Federal de São Paulo (Unifesp)
**Ana Beatriz Alvarez Perez** - Universidade Federal de São Paulo (Unifesp)
**Antonio José Gonçalves** – Faculdade de Ciências Médicas da Santa Casa de São Paulo (FCMSCSP)
**Antonio de Pádua Furquim Bonatelli** - Universidade Federal de São Paulo (Unifesp)
**Aytan Miranda Sipahi** – Hospital das Clínicas da Faculdade de Medicina da Universidade de São Paulo (HCFMUSP)
**Celso Mitsushi Massumoto** - Hospital Sírio-Libanês
**Cristina Muccioli** – Universidade Federal de São Paulo (Unifesp)
**Décio Brunoni** - Universidade Federal de São Paulo (Unifesp)
**Delcio Matos** – Universidade Federal de São Paulo (Unifesp)
**Domingo Marcolino Braile** - Faculdade de Medicina de São José do Rio Preto (Famerp) and Faculdade de Ciências Médicas da Universidade Estadual de Campinas (FCM-Unicamp)
**Edimar Alcides Bocchi** - Hospital das Clínicas da Faculdade de Medicina da Universidade de São Paulo (HCFMUSP)
**Edina Mariko Koga da Silva** – Universidade Federal de São Paulo (Unifesp)
**Edmund Chada Baracat** – Faculdade de Medicina da Universidade de São Paulo (FMUSP)
**Egberto Gaspar de Moura -** Universidade do Estado do Rio de Janeiro (UERJ)
**Eleonora Menicucci de Oliveira** - Universidade Federal de São Paulo (Unifesp)
**Elizabete Ribeiro Barros** - Universidade Federal de São Paulo (Unifesp)
**Emmanuel de Almeida Burdmann** – Faculdade de Medicina da Universidade de São Paulo (FMUSP)
**Fernando Antonio de Almeida** - Faculdade de Ciências Médicas e da Saúde (FCMS), Pontifícia Universidade Católica de São Paulo (PUC-SP)
**Fernando Ferreira Costa -** Faculdade de Ciências Médicas da Universidade Estadual de Campinas (FCM/Unicamp).
**Gaspar de Jesus Lopes Filho** - Universidade Federal de São Paulo (Unifesp)
**Gilmar Fernandes do Prado** - Universidade Federal de São Paulo (Unifesp)
**Heráclito Barbosa de Carvalho** - Faculdade de Medicina da Universidade de São Paulo (FMUSP)
**Hernani Pinto de Lemos Júnior** - Universidade Federal de São Paulo (Unifesp)
**Ioannis Minas Liontakis** - Hospital das Clínicas da Faculdade de Medicina da Universidade de São Paulo (HCFMUSP)
**Ivan Dunshee de Abranches Oliveira Santos** - Universidade Federal de São Paulo (Unifesp)
**Jacob Szejnfeld** - Universidade Federal de São Paulo (Unifesp)
**João Hamilton Romaldini** - Instituto de Assistência Médica ao Servidor Público Estadual and Pontifícia Universidade Católica de Campinas (PUC)
**José Carlos Costa Baptista-Silva** - Universidade Federal de São Paulo (Unifesp)
**José Mauro Kutner** - Hospital Israelita Albert Einstein (HIAE)
**José Roberto Filassi** - Faculdade de Medicina da Universidade de São Paulo (FMUSP)
**José Roberto Lapa e Silva** – Universidade Federal do Rio de Janeiro (UFRJ)
**José Salvador Rodrigues de Oliveira** - Universidade Federal de São Paulo (Unifesp)
**Jorge dos Santos Silva** – Hospital das Clínicas da Faculdade de Medicina da Universidade de São Paulo (HCFMUSP)
**Julio César Rodrigues Pereira -** Faculdade de Saúde Pública (FSP), Universidade de São Paulo (USP)
**Laércio Joel Franco** – Faculdade de Medicina de Ribeirão Preto (FMRP)
**Lana Maria Aguiar** - Hospital das Clínicas da Faculdade de Medicina da Universidade de São Paulo (HCFMUSP)
**Lenine Garcia Brandão** - Faculdade de Medicina da Universidade de São Paulo (HCFMUSP)
**Luiz Antônio Del Ciampo** - Faculdade de Medicina de Ribeirão Preto (FMRP)
**Luiz Henrique Gebrim** - Universidade Federal de São Paulo (Unifesp)
**Luiz Jacintho Silva** - Faculdade de Ciências Médicas da Universidade Estadual de Campinas (FCM-Unicamp)
**Marcelo Litvoc** - Hospital das Clínicas da Faculdade de Medicina da Universidade de São Paulo (HCFMUSP)
**Maria do Patrocínio Tenório Nunes** - Faculdade de Medicina da Universidade de São Paulo (FMUSP)
**Maria Regina Torloni** - Universidade Federal de São Paulo (Unifesp)
**Maricy Tacla Alves Barbosa** - Hospital das Clínicas da Faculdade de Medicina da Universidade de São Paulo (HCFMUSP)
**Marilza Vieira Cunha Rudge** – Faculdade de Medicina de Botucatu (FMB), Universidade Estadual Paulista (Unesp)
**Miguel Angelo Hyppolito -** Faculdade de Medicina de Ribeirão Preto (FMRP)
**Moacir Fernandes Godoy** - Faculdade de Medicina de São José do Rio Preto (FAMERP)
**Olavo Pires de Camargo** – Faculdade de Medicina da Universidade de São Paulo (FMUSP)
**Orlando César de Oliveira Barretto** - Faculdade de Medicina da Universidade de São Paulo (FMUSP)
**Paola Zucchi** - Universidade Federal de São Paulo (Unifesp)
**Paulo Feldner** - Universidade Federal de São Paulo (Unifesp)
**Rachel Riera** - Universidade Federal de São Paulo (Unifesp)
**Ricardo Mingarini Terra** – Hospital das Clínicas da Faculdade de Medicina da Universidade de São Paulo (HCFMUSP)
**Rozana Ciconelli** - Universidade Federal de São Paulo (Unifesp)
**Sergio Tufik** - Universidade Federal de São Paulo (Unifesp)
**Túlio Diniz Fernandes** - Faculdade de Medicina da Universidade de São Paulo (FMUSP)
**Vera Therezinha Medeiros Borges** - Universidade Estadual Paulista (Unesp)
**Vilmar Marques de Oliveira** – Faculdade de Medicina da Santa Casa de São Paulo (FMSCSP)
**Vilmon de Freitas** – Universidade Federal de São Paulo (Unifesp)
**Victor Bunduki** - Hospital das Clínicas da Faculdade de Medicina da Universidade de São Paulo (HCFMUSP)
**Walter José Gomes** – Universidade Federal de São Paulo (Unifesp)
**Willian Carlos Nahas** - Faculdade de Medicina da Universidade de São Paulo (FMUSP)
**Wilson Maça Yuki Ariê** - Faculdade de Medicina da Universidade de São Paulo (FMUSP)
**Wilson Roberto Catapani** - Faculdade de Medicina do ABC (FMABC)
**Wilson Roberto Oliveira Milani** - Hospital Sírio-Libanês (HSL)

